# Versorgung von Patienten mit Messerstichverletzungen in einem deutschen Level-1-Traumazentrum: Eine retrospektive Analyse über einen 3-Jahres-Zeitraum

**DOI:** 10.1007/s00113-026-01693-z

**Published:** 2026-03-11

**Authors:** Ludwig Jägerhuber, Georg Siebenbürger, Jan Bruder, Pascal Martin, Felix Maßen, Wolfgang Böcker, Markus Wörnle, Mareen Braunstein

**Affiliations:** 1 Klinik für Orthopädie und Unfallchirurgie, Muskuloskelettales Universitätszentrum München (MUM) LMU Klinikum, LMU München, München, Deutschland; 2 Zentrale Notaufnahme und Notaufnahmestation LMU Innenstadt, Klinik für Orthopädie und Unfallchirurgie, Muskuloskelettales Universitätszentrum München (MUM), LMU Klinikum, LMU München, München, Deutschland

**Keywords:** Stichverletzungen, Penetrierendes Trauma, Chirurgische Notfallmaßnahme, Verletzungsschwere, Thoraxtrauma, Stab wounds, Penetrating trauma, Emergency surgery, Injury severity, Thoracic trauma

## Abstract

**Hintergrund:**

Fremdzugeführte Stichverletzungen durch Messer nehmen in Deutschland sowohl in der öffentlichen Wahrnehmung als auch im klinischen Kontext an Bedeutung zu.

**Zielsetzung:**

Retrospektive Erfassung von Inzidenz, Verletzungsschwere, anatomischer Lokalisation und klinischem Verlauf von Messerstichverletzungen, die in einem universitären Maximalversorger über einen Zeitraum von 3 Jahren behandelt wurden.

**Material und Methoden:**

Eingeschlossen wurden alle Patienten, die zwischen September 2021 und August 2024 aufgrund von Messerstichverletzungen in der Notaufnahme des LMU Klinikum Innenstadt behandelt wurden. Erhoben wurden demografische Daten, Verletzungsmuster, therapeutische Maßnahmen sowie klinische Outcomes.

**Ergebnisse:**

Es wurden 48 Fälle von Messerstichverletzungen dokumentiert (47 Männer, eine Frau; mittleres Alter 36,6 Jahre, Median 27 Jahre). Am häufigsten betroffen waren die obere Extremität (25 %) und der Thorax (20,3 %). Stationär aufgenommen wurden rund 41 % der Patienten, bei 14,6 % lagen schwere Verletzungen (AIS ≥ 3) vor. Ein Patient verstarb aufgrund seiner Verletzungen.

**Diskussion:**

Obgleich selten, stellen Messerstichverletzungen ein klinisch bedeutsames Verletzungsmuster dar. Die Ergebnisse unterstreichen die Notwendigkeit einer strukturierten interdisziplinären Akutversorgung sowie die Bedeutung gezielter Vorbereitung und Trainingsmaßnahmen.

**Graphic abstract:**

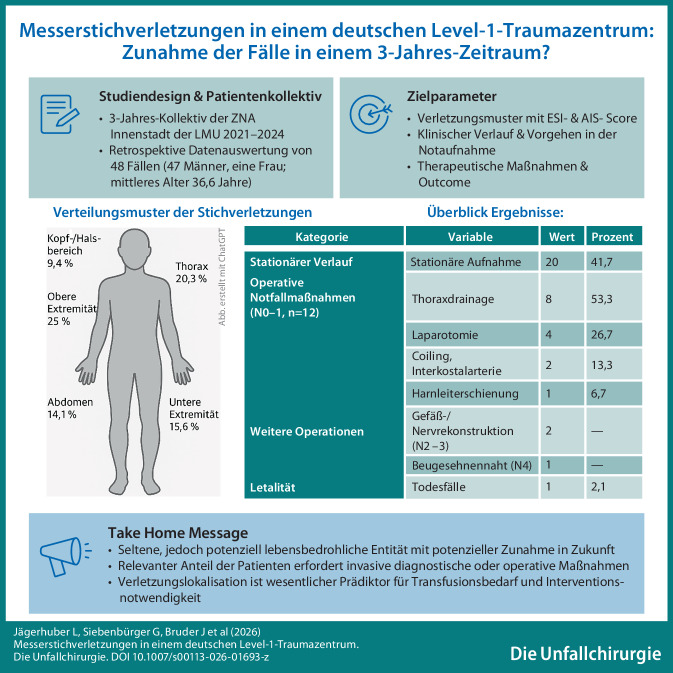

## Abstract (en)

**Background:**

Stab wounds inflicted by a third party represent an increasingly relevant form of penetrating trauma in Germany, gaining attention both in the public sphere and in clinical emergency care.

**Objective:**

This retrospective study investigated the incidence, injury severity, anatomical distribution, and clinical outcomes of stab wound cases treated at a German level I trauma center over a 3-year period.

**Material and methods:**

All patients presenting with stab injuries to the inner city emergency department of LMU University Hospital between September 2021 and August 2024 were included. A structured analysis of demographic data, injury characteristics, therapeutic interventions and clinical outcomes was conducted.

**Results:**

A total of 48 cases were identified (47 male, 1 female; mean age 36.6 years, median 27 years). The most frequently affected anatomical regions were the upper extremities (25%) and thorax (20.3%). Hospital admission was required in 41.7% of cases, 14.6% sustained major trauma (abbreviated injury scale, AIS ≥ 3) and 1 patient died.

**Discussion:**

Stab wounds continue to pose a rare yet clinically significant challenge. The findings underline the necessity for structured interdisciplinary management and targeted emergency preparedness protocols in trauma systems.

## Einleitung

Penetrierende Verletzungen stellen in Deutschland insgesamt ein seltenes, jedoch klinisch hochrelevantes Verletzungsmuster dar. Im Vergleich zu stumpfen Traumata gehen sie mit einer erhöhten Rate an Organverletzungen, einem höheren Risiko lebensbedrohlicher Blutungen sowie einer gesteigerten Notwendigkeit notfallchirurgischer Interventionen einher [7]. Insbesondere Messerstichverletzungen erfordern aufgrund ihrer Unvorhersehbarkeit, der häufig eingeschränkten klinischen Einschätzbarkeit der Verletzungstiefe und der potenziell raschen Dynamik eine strukturierte und interdisziplinäre Akutversorgung [13].

Während in Ländern mit hoher Inzidenz penetrierender Traumata – etwa in Teilen der Vereinigten Staaten von Amerika (USA) oder Südafrikas umfangreiche klinische und epidemiologische Daten vorliegen, ist die Studienlage in Deutschland weiterhin limitiert [12, 21]. Verfügbare Daten stammen überwiegend aus polizeilichen Kriminalstatistiken oder Registeranalysen, die zwar Rückschlüsse auf Häufigkeit und Umstände zulassen, jedoch nur eingeschränkt Aussagen zur tatsächlichen Verletzungsschwere, zum klinischen Verlauf und zu therapeutischen Konsequenzen ermöglichen.

Für die unfallchirurgische Versorgung bedeutet dies, dass Messerstichverletzungen trotz ihrer Seltenheit jederzeit mit potenziell lebensbedrohlicher Konsequenz auftreten können, während gleichzeitig eine geringe Fallzahl die Ausbildung von Routine erschwert. Diese Kombination aus niedriger Inzidenz und hoher klinischer Relevanz stellt besondere Anforderungen an Strukturqualität, Prozessabläufe und interdisziplinäre Zusammenarbeit im Schockraum.

Ziel der vorliegenden Arbeit war es daher, fremdbeigebrachte Messerstichverletzungen, die in der Zentralen Notaufnahme eines universitären Maximalversorgers behandelt wurden, systematisch zu erfassen und hinsichtlich Verletzungsmuster, Versorgungsdringlichkeit, therapeutischer Maßnahmen und des klinischen Verlaufs zu analysieren. Durch die Aufarbeitung eines klinischen Kollektivs soll ein Beitrag zur realistischen Einschätzung dieser Verletzungsentität in der deutschen Versorgungslandschaft geleistet und eine Grundlage für gezielte Ausbildungs- und Trainingskonzepte geschaffen werden.

## Material und Methoden

Die vorliegende Arbeit ist eine retrospektive Beobachtungsstudie, die im Zeitraum vom 01.09.2021 bis 31.12.2024 in der Zentralen Notaufnahme (ZNA) des Universitätsklinikums der Ludwig-Maximilians-Universität (LMU) am Campus Innenstadt in München durchgeführt wurde. Ein positives Ethikvotum der Ethikkommission der LMU liegt unter der Nummer AZ 25-0109 vor. Eingeschlossen wurden alle Patienten, die im oben genannten Zeitraum aufgrund einer fremdzugeführten Messerstichverletzung in der ZNA behandelt wurden. Die Datenerhebung erfolgte unter Nutzung des klinikinternen IT-Systems der Notaufnahme (EPIAS®, epias GmbH, Idstein, Germany) sowie des Krankenhausinformationssystems (KIS/SAP). Erfasst wurden u. a. Alter, Geschlecht und Nationalität der Patienten. Präklinisch erfolgte die Einschätzung der Verletzung nach dem National Advisory Committee for Aeronautics (NACA) und innerklinisch anhand des Emergency Severity Index (ESI) [8–10]. Zur objektiven Bewertung der Verletzungsschwere wurde die Abbreviated Injury Scale (AIS) herangezogen [22]. Infobox 1 gibt eine strukturierte Übersicht über die weiteren erhobenen Variablen.

### Infobox Übersicht der erhobenen prä- und innerklinischen Parameter

- Patientenalter
- Geschlecht
- Datum und Uhrzeit der Vorstellung
- Schockraumaufnahme (ja/nein)
- Verletzungsmuster
- Verletzungslokalisation
- Notfallmaßnahmen
- Überleben
- NACA-Score
- Triagierung, ESI-Score
- AIS-Score
- Befund des Focused Assessment with Sonography for Trauma (FAST)
- Operationsindikation/-befund
- Intensivstationsaufenthalt
- EK-Transfusion
- Abschlussdiagnosen

## Statistische Auswertung

Kategoriale Variablen werden als absolute Häufigkeiten (*n*) und relative Anteile (%) angegeben, kontinuierliche Variablen als arithmetische Mittelwert sowie Median und Interquartilsabstand (IQR) sowie deskriptive Statistiken. Ein Gruppenvergleich ist bei der sehr ungleichen Geschlechtsverteilung nicht sinnvoll.

Alle Patientendaten wurden vollständig anonymisiert, und eine Reidentifikation ist ausgeschlossen. Die Analyse erfolgte unter strikter Einhaltung der Vorgaben der Datenschutz-Grundverordnung (DSGVO).

## Ergebnisse

### Demografische Daten

**Abb. 1:**
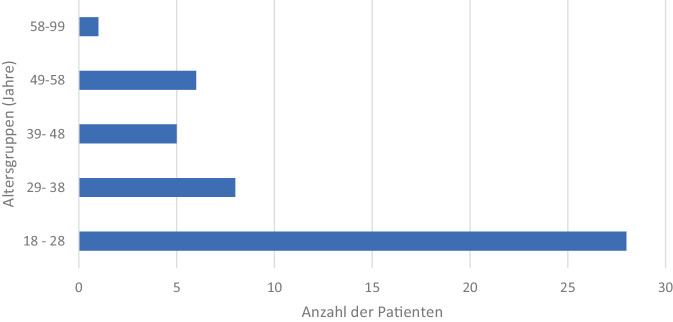
Altersverteilung der Patient*innen

Im Beobachtungszeitraum wurden 48 Patienten (0,01 %) mit fremdzugeführten Messerstichverletzungen bei insgesamt rund 64.800 chirurgischen Patientenkontakten in der ZNA behandelt. 47 Personen waren männlich, eine Person weiblich. Das Alter lag zwischen 18 und 60 Jahren mit einem durchschnittlichen Alter von 36,6 Jahren (Median 27 Jahre, IQR 16 Jahre; Abb. 1).

**Abb. 2:**
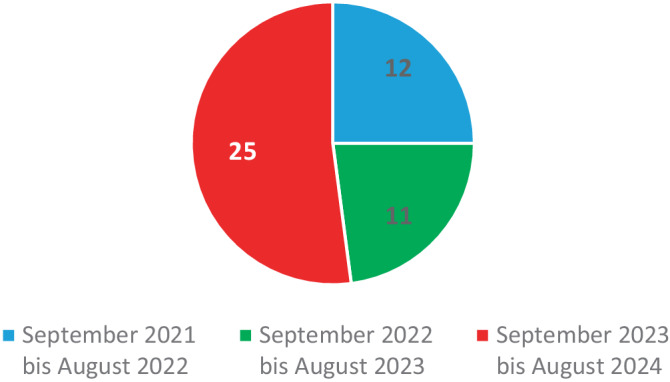
Verteilung der Vorstellungen der Patienten innerhalb des 3‑Jahres-Zeitraums in der ZNA

Im Zeitraum September 2021 bis August 2022 wurden insgesamt 12 Patienten behandelt. Von September 2022 bis August 2023 waren es 11 Fälle und von September 2023 bis August 2024 wurden 25 Fälle registriert (Abb. 2).

**Abb. 3:**
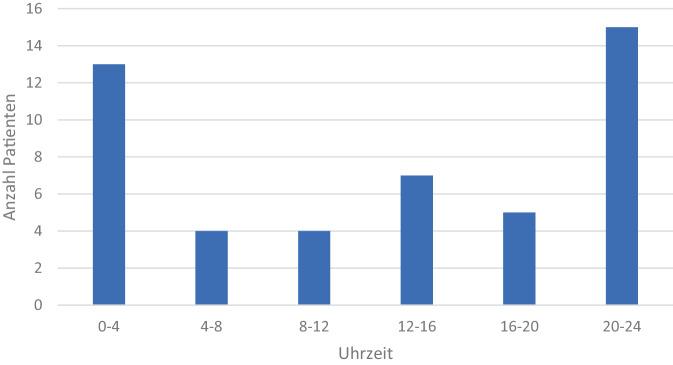
Tageszeitliche Verteilung der Vorstellung in der ZNA nach Uhrzeitgruppierung

Die meisten Behandlungen erfolgten am Freitag bzw. am Montag (*n* = 10), gefolgt von Mittwoch bzw. Donnerstag (jeweils *n* = 8) und Dienstag (*n* = 7). Samstage (*n* = 3) und Sonntage (*n* = 2) zeigten eine geringere Häufung. Der höchste Anteil der Fälle (*n* = 15; 31,3 %) fiel in den Zeitraum zwischen 20:00 und 24:00 Uhr. Die weiteren Zeiträume verdeutlicht Abb. 3.

### Präklinische Daten

Die präklinische Einschätzung der Verletzungsschwere erfolgte anhand des NACA-Scores durch den Notarzt oder Rettungsdienst. Dabei wurden 19 Fälle (39,6 %) der NACA-Kategorie III (mäßige bis schwere Störungen ohne akute Lebensbedrohung) zugeordnet. Zehn Patienten (20,8 %) wurden als NACA IV (notärztliche Versorgung erforderlich), 18 Fälle (37,5 %) als NACA V (Transport unter Reanimationsbereitschaft) klassifiziert. Ein Patient (2,1 %) wurde unter laufender kardiopulmonaler Reanimation aufgenommen (NACA VI). Präklinisch wurde in keinem Fall eine invasive Maßnahme wie das Anlegen einer Thoraxdrainage oder die Applikation eines Tourniquets dokumentiert.

### Innerklinische Ersteinschätzung (Triage)

Die Ersteinschätzung in der Notaufnahme erfolgte nach dem ESI-Score. Insgesamt 19 Patienten (39,6 %) wurden im Schockraum versorgt (ESI 1). Weitere 10 Fälle (20,8 %) wurden mit ESI 2, 12 Fälle (25,0 %) mit ESI 3 und 7 Fälle (14,6 %) mit ESI 4 triagiert.

### Verletzungsschwere

Die Analyse der Verletzungsschwere anhand des AIS-Scores ergab, dass bei 40 Patienten (70,2 %) ein Score von 1 dokumentiert wurde. Drei Fälle (5,3 %) wiesen einen AIS-Score von 2 und 9 Fälle (15,8 %) einen AIS-Score von 3 auf. Ein AIS-Score der Schweregradstufe 4 lag in 5 Fällen (8,8 %) vor. In 13 Fällen (27,1 %) lag eine Mehrfachverletzung vor (Tab. 1).

**Tab. 1:** Übersicht zu Demografie, Verletzungsschwere, Lokalisation und Behandlung bei Messerstichverletzungen

Kategorie	Variable	Wert	Prozent (%)
*Patientencharakteristika*	Gesamtzahl, Patienten	48	100,0
	Männlich	47	97,9
	Weiblich	1	2,1
	Altersbereich (Jahre)	18–60	–
	Median	27	–
	IQR	16	–
*Präklinische Einschätzung (NACA)*	NACA III	19	39,6
	NACA IV	10	20,8
	NACA V	18	37,5
	NACA VI	1	2,1
*Ersteinschätzung, Notaufnahme (ESI)*	ESI 1	19	39,6
	ESI 2	10	20,8
	ESI 3	12	25,0
	ESI 4	7	14,6
*AIS-Score*	AIS 1	40	70,2
	AIS 2	3	5,3
	AIS 3	9	15,8
	AIS 4	5	8,8
*Verletzungslokalisation*	Obere Extremität	16	25,0
	Thorax	13	20,3
	Untere Extremität	10	15,6
	Abdomen	9	14,1
	Kopf‑/Halsbereich	6	9,4
*Weitere Befunde*	Freie intraabdominelle Flüssigkeit (FAST)	2	4,2
*Stationärer Verlauf*	Stationäre Aufnahme	20	41,7
*Transfusion innerhalb 24* *h*	1 EK	1	–
	2 EK	2	–
	3 EK	1	–
	6 EK	1	–
	8 EK	1	–
	11 EK	1	–
*Operative Notfallmaßnahmen (N0–1, n* *=* *12)*	Thoraxdrainage	8	53,3
	Laparotomie	4	26,7
	Coiling, Interkostalarterie	2	13,3
	Harnleiterschienung	1	6,7
*Weitere Operationen*	Gefäß‑/Nervrekonstruktion (N2–3)	2	–
	Beugesehnennaht (N4)	1	–
*Letalität*	Todesfälle	1	2,1

### Verletzungslokalisation

**Abb. 4:**
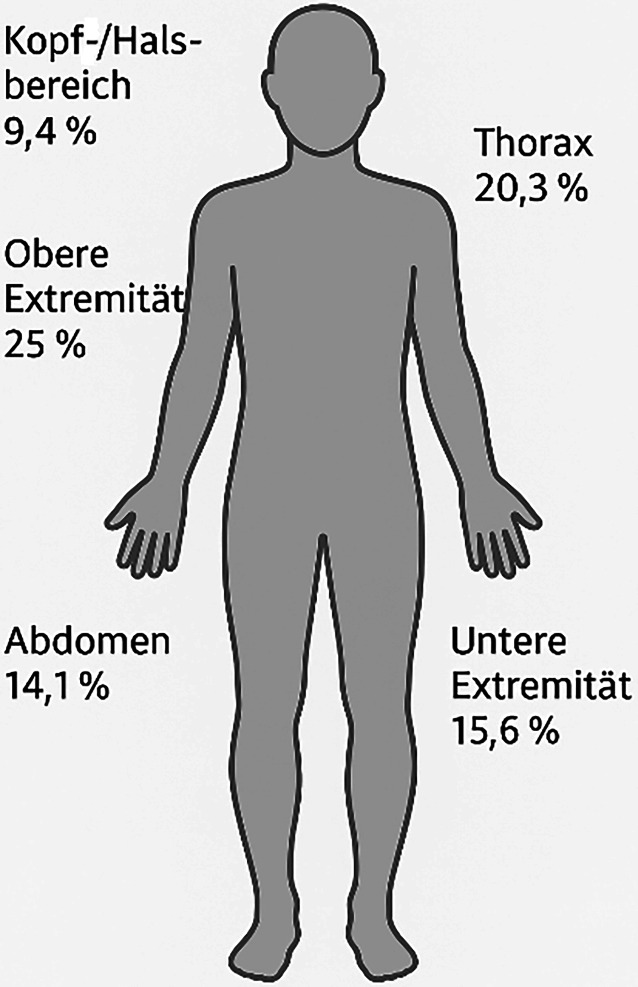
Verteilungsmuster der Stichverletzungen (erstellt mithilfe von chatGPT)

Die häufigsten Verletzungen traten mit 16 Fällen (25 %) an der oberen Extremität auf. 13 Fälle (20,3 %) wiesen Verletzungen im Bereich des Thorax und 10 Fälle (15,6 %) an der unteren Extremität auf. Verletzungen im Bereich des Abdomens traten in 9 Fällen (14,1 %) und des Kopf‑/Halsbereiches in 6 Fällen (9,4 %) auf (Abb. 4).

### Klinischer Verlauf

Zwei Patienten (4,2 %) zeigten in der initial durchgeführten FAST-Sonographie Hinweise auf freie intraabdominelle Flüssigkeit. 20 von 48 Patienten (41,7 %) wurden zur stationären Behandlung aufgenommen. Innerhalb der ersten 24 h nach Aufnahme erhielten 7 Patienten eine Transfusion von EK. Die verabreichten Transfusionsmengen verteilten sich wie folgt: einmal 1 EK, zweimal 2 EK, einmal 3 EK, einmal 6 EK, einmal 8 EK und einmal 11 EK, wobei nur bei Thorax- (*n* = 4) und Abdomenverletzungen (*n* = 3) transfundiert wurde. Ein Patient verstarb im Rahmen des stationären Verlaufs, was einer Letalitätsrate von 2,1 % im Beobachtungskollektiv entspricht.

### Interventionelle/operative Versorgung

Zwölf Patienten wurden in die Kategorien N0–1 eingestuft, wobei die häufigsten operativen Notfallmaßnahmen Thoraxdrainagen (66,7 %) und Laparotomien (33,3 %) waren. In 2 Fällen erfolgte eine Naht eines verletzten Darmsegments. Ein Patient verstarb unmittelbar postoperativ nach einer Laparotomie im hämorrhagischen Schock.

Es wurden 2 notfallmäßige Coiling-Verfahren bei Blutungen aus den Interkostalarterien und in je einem Fall eine Thorakoskopie und eine Harnleiterschienung durchgeführt.

In der Kategorie N2–3 gab es 2 Fälle von Gefäß- bzw. Nervendurchtrennung mit anschließender Rekonstruktion. Bei einer N4-Operation handelte es sich um eine Beugesehnennaht.

## Diskussion

Penetrierende Verletzungen durch Messer stellen in Deutschland weiterhin eine seltene, jedoch klinisch hochrelevante Entität dar. Die vorliegende retrospektive Analyse eines universitären Maximalversorgers liefert einen differenzierten Einblick in Verletzungsmuster, Versorgungsdringlichkeit und therapeutische Konsequenzen fremdbeigebrachter Messerstichverletzungen über einen Zeitraum von 3 Jahren. Ziel war es, die klinische Realität dieser Verletzungsform jenseits kriminalstatistischer Erhebungen abzubilden und deren Bedeutung für die unfallchirurgische Akutversorgung einzuordnen.

Eine valide Einschätzung der tatsächlichen Verletzungsschwere sowie der daraus resultierenden klinischen Konsequenzen ist nur durch systematische klinische Erhebungen und epidemiologische Analysen möglich. Diese Differenzierung ist von erheblicher klinischer Bedeutung, da penetrierende Thoraxverletzungen im Vergleich zu stumpfen Thoraxtraumata mit einer bis zu 30 % höheren Inzidenz invasiver notfallmedizinischer Maßnahmen, einschließlich operativer Interventionen, assoziiert sind [25, 31].

### Demografische Daten

Das untersuchte Kollektiv zeigte eine ausgeprägte Geschlechterdisparität mit überwiegendem Anteil junger männlicher Patienten, was mit nationalen Registerdaten sowie internationalen Studien übereinstimmt [2, 6, 15, 20]. Die Zahlen sind denen des TraumaRegister DGU® (TR-DGU) im Zeitraum von 2009 bis 2018 mit einem durchschnittlichen Alter von 39 ± 17 Jahren und hohem Männeranteil von 84 % ähnlich [7]. Auch die aktuelle GewPen-Studie aus Düsseldorf dokumentierte ein sehr ähnliches Kollektiv (Männer: 79,3 %; Altersdurchschnitt 33 ± 14 Jahre).

### Fallzahlen und tageszeitlicher Verlauf

Im Beobachtungszeitraum zeigte sich im letzten Beobachtungsdrittel eine höhere Fallzahl im Vergleich zu den beiden Zeiträumen. Aufgrund der geringen absoluten Zahlen und des kurzen Beobachtungszeitraums ist jedoch keine belastbare Trendanalyse möglich. Bei Fokussierung auf die Fragestellung von fremdzugeführten Messerstichverletzungen wurden selbstbeigebrachte Verletzungen nicht berücksichtigt, was Einschränkungen in der Generalisierbarkeit bedeutet. Die beobachtete Zunahme ist daher vorsichtig zu interpretieren und kann ebenso zufälligen Schwankungen entsprechen. Aussagen über eine generelle Inzidenzsteigerung lassen sich aus diesen Daten nicht ableiten.

Im Allgemeinen zeigt der aktuelle Jahresbericht 2024 des TraumaRegister DGU® mit 1278 Fällen einen Anstieg der Fälle mit Messerstichverletzungen im Vergleich zu den Vorjahren auf; ähnlich verhält es sich in Großbritannien mit seit 3 Jahren steigender Inzidenz [28]. Die zentrale Lage der Notaufnahme im Umfeld des Münchner Hauptbahnhofs, an dem bundesweit die meisten Gewaltdelikte an einem Bahnhof registriert wurden, hier 735 Gewaltdelikte gefolgt vom Berliner Hauptbahnhof mit 715 und dem Kölner Hauptbahnhof mit 682, erklärt die Situation nur bedingt [14]. Ebenso stellt die Nachbarschaft mit bekanntem Drogen- und Konflikthotspots einen plausiblen Kontextfaktor dar, erlaubt jedoch auf Basis der vorliegenden Daten keine kausale Zuordnung des beobachteten Anstiegs. Nach Angaben der Stadt häuften sich Körperverletzungen und Drogendelikte zunehmend, sodass die Stadt München mit einem Waffen‑/Messer- und Alkoholverbot ab 15.01.2025 in diesen Bereichen reagierte [1].

Die meisten Aufnahmen traten zwischen 20:00 und 4:00 Uhr auf, was mit dem erhöhten Risiko für Gewalt und Auseinandersetzungen in den Abendstunden übereinstimmt [15]. Auch Schürmann et al. berichten über die Hälfte aller Aufnahmen (55 %) in den Abend- und Nachtstunden sowie 43 % der Einsätze an Wochenendtagen. In unserem Kollektiv erfolgten die Aufnahmen gehäuft an Freitagen und Montagen. Dies deckt sich nur z. T. mit der Literatur, welche v. a. Freitag bis Sonntag eine Häufung beschreibt. Die im vorliegenden Kollektiv beobachtete Häufung an Montagen sowie der relative Abfall an Samstagen weichen von anderen epidemiologischen Erhebungen ab. Aufgrund der geringen Fallzahlen ist hier am ehesten von zufälligen Variationen auszugehen, sodass eine belastbare Interpretation und generalisierbare Aussagen eingeschränkt sind.

### Präklinische Einschätzung und Versorgungsstrategien

Die präklinische Einschätzung anhand des NACA-Scores wies einen hohen Anteil potentiell schwerer Verletzungen auf. Bemerkenswert ist, dass trotz dieser Einstufung in keinem Fall präklinisch invasive Maßnahmen wie Thoraxdrainagen oder Tourniquet-Anlagen dokumentiert wurden. Dies lässt sich am ehesten durch die kurzen Transportzeiten im innerstädtischen Bereich sowie durch ein „scoop-and-run“-orientiertes Versorgungskonzept erklären, bei dem der schnelle Transport in ein Traumazentrum priorisiert wird [4, 27]. Regionale Rettungsdienstalgorithmen sehen bei penetrierenden Verletzungen häufig eine zügige innerklinische Versorgung vor. Eine relevante präklinische Unterversorgung erscheint vor dem Hintergrund der guten klinischen Outcomes und der raschen innerklinischen Versorgung eher unwahrscheinlich. Dennoch unterstreichen die Daten die Bedeutung regionaler Einsatzkonzepte und deren Einfluss auf das präklinische Interventionsspektrum.

### Triage

In der untersuchten Kohorte wurden knapp 40 % der Patientinnen und Patienten mit der höchsten Dringlichkeitsstufe im ESI-Score eingestuft, gleichzeitig wiesen viele Patienten eine niedrige objektive Verletzungsschwere nach dem AIS auf. In der statistischen Auswertung zwischen der ESI-Triage und der objektiven Verletzungsschwere nach AIS zeigt sich jedoch nur ein moderater Zusammenhang (Pearsons r = 0,47). Diese Diskrepanz verdeutlicht die Limitation beider Systeme: Während der ESI primär die Dringlichkeit und den zu erwartenden Ressourcenbedarf abbildet, bewertet der AIS die anatomische Verletzungsschwere. Gerade bei penetrierenden Verletzungen ist initial häufig keine sichere Abschätzung der Verletzungstiefe und Organbeteiligung möglich, sodass eine hohe Triage-Stufe gerechtfertigt ist. Die Ergebnisse unterstreichen, dass die initiale Einschätzung bei Penetrationstraumata bewusst großzügig erfolgen sollte, um potenziell lebensbedrohliche Verläufe nicht zu übersehen.

### Verletzungsmuster

Die meisten Verletzungen betrafen die obere Extremität und den Thorax, was ein sehr häufiger Mechanismus bei Auseinandersetzungen mit körperlicher Gewalt und konsekutiven Abwehrmechanismen ist [16, 17]. Da penetrierende Thoraxverletzungen im Vergleich zu stumpfen Thoraxtraumata mit einer bis zu 30 % höheren Inzidenz invasiver notfallmedizinischer Maßnahmen, einschließlich operativer Interventionen, assoziiert sind, ist dies hinsichtlich dringender invasiven Maßnahmen relevant [23, 25, 31]. Diese Daten decken sich mit unseren Ergebnissen der prädominierenden Verletzungen im Bereich von Thorax und Abdomen, wobei penetrierende Verletzungen, insbesondere die Zweihöhlenverletzungen, mit einem deutlich erhöhtem Volumenmangel, einer vermehrten Notwendigkeit von Blutprodukttransfusionen sowie erhöhten Mortalität und einer Letalität von rund 45 % einhergehen [7, 18, 29, 30]. Im vorliegenden Kollektiv wiesen alle transfundierten Patienten Verletzungen des Körperstamms auf, überwiegend thorakal und/oder abdominal. Isolierte Extremitätenverletzungen führten in keinem Fall zur Transfusion. In Analysen zeigten, dass in 15 % der Todesfälle potenziell bzw. tatsächlich vermeidbare Todesursachen – wie Perikardtamponade, Hämatopneumothorax oder Spannungspneumothorax, dringend indizierte, lebensrettende Maßnahmen zu spät oder nicht durchgeführt worden, wobei in nur 5 % der Fälle eine prähospitale Thoraxdrainagenanlage erfolgte [7, 19]. Diese unterstreicht die prognostische Bedeutung der Verletzungslokalisation bei penetrierenden Traumata insbesondere im Körperstammbereich.

### Operative und interventionelle Versorgung

Die Mehrheit unserer Patienten (70,2 %) wies nur geringe körperliche Verletzungen nach dem AIS-Score auf. Gleichzeitig war jeder vierte Patienten (24,6 %) als schwerwiegend verletzt einzustufen, und in rund 25 % der Fälle lag eine Mehrfachverletzung vor. Rund 40 % der Patienten wurden über den Schockraum vorgestellt, und der AIS-Score ordnet 14 Patienten (24,6 %) in die 2 höchsten Gruppen ein. Dies deckt sich mit den Daten der GewPen-Studie [26]. Ein relevanter Anteil der Patienten erforderte notfallchirurgische oder interventionelle Maßnahmen, insbesondere Thoraxdrainagen, Laparotomien sowie in Einzelfällen interventionelle Coiling-Verfahren. Diese Eingriffe stellen klassische Beispiele für seltene, jedoch zeitkritische Maßnahmen dar, die im Schockraumsetting sicher beherrscht werden müssen, da penetrierende Verletzungen jederzeit hochakute Interventionsnotwendigkeiten erzeugen können [3, 11].

### Letalität

Unsere Arbeit hat lediglich Patienten eingeschlossen, die lebend die Notaufnahme erreicht haben. Die Düsseldorfer Kollegen berichten von einer Inzidenz von Todesfällen an der Einsatzstelle von rund 1 %, wobei in unserem Studienkollektiv die innerklinischen Letalität mit 2 % vergleichsweise gering war. Hier erschient die Letalität penetrierender Verletzungen im internationalen Vergleich mit angegebenen Letalitätsraten bis zu 15 % niedrig [24].

### Internationaler Vergleich

Im internationalen Vergleich, insbesondere zu Hochinzidenzregionen wie Südafrika oder bestimmten urbanen Zentren Großbritanniens, sind penetrierende Traumata in Deutschland deutlich seltener [21]. Während dort Messerstichverletzungen einen relevanten Anteil des täglichen Traumageschehens darstellen, bleiben sie in deutschen Traumazentren eine seltene, jedoch klinisch hochrelevante Entität. Die Verletzungsmuster – insbesondere die hohe Rate thorakaler und abdomineller Beteiligung – sind jedoch vergleichbar [5].

Die insgesamt niedrige Letalität in unserem Kollektiv dürfte durch den schnellen Zugang zu hochspezialisierter Traumaversorgung, strukturierte Schockraumprozesse und interdisziplinäre Zusammenarbeit erklärbar sein [7]. Unterschiede in präklinischen Interventionsraten weisen zudem auf strukturelle Unterschiede der Rettungsdienstsysteme hin.

### Einordnung in das Gesamtkollektiv der Notaufnahme

Im Verhältnis zum gesamten chirurgischen Notfallaufkommen unserer Zentralen Notaufnahme stellen Messerstichverletzungen, wie erwartet, weiterhin einen sehr geringen Anteil dar. Sie sind somit seltene Ereignisse, zeichnen sich jedoch durch ein hohes potenzielles Schadensausmaß und eine besondere Dynamik aus. Diese Kombination aus geringer Inzidenz und hoher klinischer Relevanz stellt besondere Anforderungen an Ausbildung, Struktur und Prozessqualität.

### Implementierung in den klinischen Alltag

Vor dem Hintergrund der in dieser Untersuchung dokumentierten Zunahme von Messerstichverletzungen und der damit verbundenen Notwendigkeit seltener, aber potenziell lebensrettender Notfalleingriffe wurde im Dezember 2023 am Klinikum Innenstadt der LMU München erstmals ein hochrealistisches In-situ-Simulationstraining zur „Notfallthorakotomie bei penetrierender Verletzung“ durchgeführt. In Zusammenarbeit mit dem Institut für Notfallmedizin und Medizinmanagement (INM) sowie unter Nutzung der LMU-internen 3D-Druck-Infrastruktur konnte eine spezialisierte Simulationspuppe entwickelt werden, die alle relevanten Prozeduren – einschließlich Mini- und Clamshell-Thorakotomie, Perikardiotomie, offener Defibrillation, manueller Aortenkompression sowie Atemwegs- und Volumenmanagement – realitätsnah abbildet. Das in unserem Haus implementierte Simulationstraining ist interdisziplinär angelegt und erfolgt in enger Zusammenarbeit mit Personen der ZNA und OP-Pflege sowie der Anästhesie, Radiologie, Viszeral- und Thoraxchirurgie. Ziel ist nicht die Substitution fachfremder Expertise, sondern die strukturierte Vorbereitung auf initiale, zeitkritische Maßnahmen im Schockraumsetting sowie die Optimierung der Schnittstellenkommunikation. Insbesondere seltene, aber lebensrettende Eingriffe („low frequency – high stakes“) erfordern ein hohes Maß an Teamkoordination, klaren Algorithmen und Handlungssicherheit.

Bis zum Abschluss der Datenerhebung wurde jedoch noch keine Indikation zur Notfallthorakotomie gestellt. Ergänzend wurden interprofessionelle wöchentliche Fortbildungen, inklusive Gerätekunde („Equipment Friday“), etabliert. Die nachfolgenden Evaluationen zeigte eine deutliche Verbesserung des subjektiven Kompetenzempfindens. Neben der Stärkung individueller und teambezogener Handlungssicherheit trug das Training auch zur praxisnahen Optimierung theoretisch formulierter SOP und geplanter Abläufe bei.

## Limitationen

Als monozentrische, retrospektive Beobachtungsstudie ist die Aussagekraft der Ergebnisse in ihrer Generalisierbarkeit eingeschränkt. Das beobachtete Kollektiv bezieht sich ausschließlich auf eine universitäre Notaufnahme in einem städtischen Ballungsraum, was Rückschlüsse auf ländliche oder andere urbane Regionen nur bedingt zulässt. Die geringe Fallzahl limitiert die Aussagekraft statistischer Vergleiche und erlaubt keine belastbaren Trendanalysen. Zudem wurden ausschließlich fremdbeigebrachte penetrierende Verletzungen eingeschlossen, sodass keine Aussagen zu autoaggressiven Verletzungsmustern möglich sind. Auch die retrospektive Erhebung präklinischer Maßnahmen ist abhängig von der Qualität der Dokumentation.

## Schlussfolgerung

Die vorliegende Arbeit zeigt, dass fremdbeigebrachte Messerstichverletzungen in deutschen Traumazentren selten, jedoch klinisch hochrelevant sind. Trotz häufig niedriger objektiver Verletzungsschwere ist die initiale Versorgungsdringlichkeit hoch, und ein relevanter Anteil der Patienten erfordert invasive Maßnahmen. Die Ergebnisse unterstreichen die Notwendigkeit einer strukturierten, interdisziplinären Akutversorgung sowie gezielter Ausbildungs- und Trainingskonzepte zur Vorbereitung auf diese seltenen, aber potenziell lebensbedrohlichen Szenarien.

## Fazit für die Praxis

Fremdbeigebrachte Messerstichverletzungen stellen in deutschen Traumazentren eine seltene, jedoch potenziell lebensbedrohliche Entität dar. Trotz häufig niedriger äußerer Verletzungsschwere besteht eine hohe initiale Versorgungsdringlichkeit, da die tatsächliche Penetrationstiefe und Organbeteiligung präklinisch wie initial klinisch oft nicht sicher abschätzbar sind. Ein relevanter Anteil der Betroffenen erfordert invasive diagnostische oder operative Interventionen, insbesondere bei thorakaler und abdomineller Lokalisation, die als Prädiktoren für Transfusionsbedarf und Interventionsnotwendigkeit gelten.

Angesichts der niedrigen Inzidenz und hohen Dynamik sind strukturierte Schockraumkonzepte, standardisierte Alarmierungswege sowie simulationsbasiertes Training für seltene, zeitkritische Eingriffe essenziell, um Handlungssicherheit, interdisziplinäre Koordination und Versorgungsqualität nachhaltig zu gewährleisten.

## Data Availability

Alle Daten können auf Anfrage zugänglich gemacht werden.
